# Myasthenia Gravis Can Have Consequences for Pregnancy and the Developing Child

**DOI:** 10.3389/fneur.2020.00554

**Published:** 2020-06-12

**Authors:** Nils Erik Gilhus

**Affiliations:** ^1^Department of Clinical Medicine, University of Bergen, Bergen, Norway; ^2^Department of Neurology, Haukeland University Hospital, Bergen, Norway

**Keywords:** myasthenia gravis, autoimmunity, autoantibodies, pregnancy, neonatal myasthenia, arthrogryposis, breastfeeding, teratogenicity

## Abstract

Myasthenia gravis (MG) with onset below 50 years, thymic hyperplasia and acetylcholine receptor (AChR) antibodies is more common in females than in males. For a relatively large group of MG patients, pregnancy represents therefore an important question. The muscle weakness, the circulating autoantibodies, the hyperplastic thymus, the MG drug treatment, and any autoimmune comorbidity may all influence both mother and child health during pregnancy and also during breastfeeding in the postpartum period. Mother's MG remains stable in most patients during pregnancy. Pyridostigmine, prednisolone, and azathioprine are regarded as safe during pregnancy. Mycophenolate, methotrexate and cyclophosphamide are teratogenic and should not be used by women with the potential to become pregnant. Rituximab should not be given during the last few months before conception and not during pregnancy. Intravenous immunoglobulin and plasma exchange can be used for exacerbations or when need for intensified therapy. Pregnancies in MG women are usually without complications. Their fertility is near normal. Vaginal delivery is recommended. MG patients have an increased rate of Cesarean section, partly due to their muscle weakness and to avoid exhaustion, partly as a precaution that is often unnecessary. Around 10% of the newborn develop neonatal myasthenia during the first few days after birth. This is transient and usually mild with some sucking and swallowing difficulties. In rare cases, transplacental transfer of AChR antibodies leads to permanent muscle weakness in the child, and arthrogryposis with joint contractures. Repeated spontaneous abortions have been described due to AChR antibodies. MG women should always give birth at hospitals with experience in newborn intensive care. MG does not represent a reason for not having children, and the patients should be supported in their wish of becoming pregnant.

## Introduction

Myasthenia gravis (MG) is an autoimmune disorder where well-defined muscle antibodies bind to the post-synaptic membrane at the neuromuscular junction (1). These antibodies induce the muscular weakness typical for MG. In most patients, the antibodies bind to acetylcholine receptors (AChR), but alternative targets are muscle-specific tyrosine kinase (MuSK) and lipoprotein-related peptide 4 (LRP4) (2). Antibody binding leads to destruction and reduced receptor function through cross-linking of membrane molecules, complement activation, and blockade of ligand-binding epitopes. AChR antibody binding to the cell membrane leads in addition to a cascade of intracellular events that influences muscle cell function. AChR and MuSK antibodies are highly specific for MG, as they do not occur without muscle weakness and in the healthy population. MG severity is not linked to autoantibody concentration, illustrating the variation and complexity in the antigen-antibody interaction as well as the individual variation in the consequences of this interaction.

Most females in reproductive age with MG have an enlarged and hyperplastic thymus with widespread germinal follicles (3). A thymoma is present in 10% of all MG patients but is less common among young females with the disease. The thymus pathology induces the production of AChR antibodies in lymphoid tissue widespread in the body. The trigger for thymic hyperplasia is not known, but virus infection has been suggested, in genetically predisposed individuals and perhaps at an especially vulnerable time point. Thymectomy early in the disease improves MG in females in reproductive age (4). Thymic hyperplasia does not occur in other autoimmune disorders, and the therapeutic effect of thymectomy is specific for MG with AChR antibodies.

Untreated MG is a severe disease with 50% mortality after ten years. With modern treatment, no patients should die from their MG. Therapy combines symptomatic and immunosuppressive drugs, thymectomy, and supportive therapy such as physical training, vigorous treatment of infections, respiratory support in the rare occasions it becomes necessary, and optimal treatment of comorbidities. Most patients do well and have modest, minimal or no muscle weakness. However, 10–20 % have a disease that is relatively resistant to standard therapies.

MG prevalence in the general population is 150–250 individuals per million, and with an annual incidence of 8–10 individuals per million (5). As both prevalence and incidence increase with increasing age, these figures are somewhat lower among females in reproductive age. MG prevalence in European females below age 50 years is thought to be 120 per million, and annual incidence 5–10 per million (5). MG with onset below age 50 years and AChR antibodies is 2–3 times more common in females than in males, and women have an incidence peak at age 30 years. In China and other Far East countries, juvenile MG is much more common than in Western populations, and MG with debut in childhood represents a third incidence peak (6). MuSK MG is more common in older age groups. However, in a multinational study from mainly Western countries, 70% had MuSK MG debut before age 40 years, and the females had a mean debut age of 31 years (7). MuSK MG is twice as common in females as in males. The relative number of MG patients in different age groups depends for a large part on population demographics. In younger populations in Africa, South-America and Africa, pregnancy and childbirth is relevant for a much larger proportion of MG patients than in Europe.

Mother's age when giving birth has increased markedly during the last decades, especially in Western countries. In Norway, the mean age was in 2018 31 years, up from 29 years ten years ago. Similarly, mean age at first childbirth has increased from 23.5 years in 1975 to 25.5 years in 1990, and to 29.5 years in 2018 (www.ssb.no/fodte). This increased age at childbirth means that a higher proportion of MG females will experience childbirth after manifest disease.

For females in reproductive age with MG, one of their major concerns is potential consequences for fertility, pregnancy, giving birth, and lactation (8). Any risks for the child as well as for themselves are of the highest importance. Furthermore, they would like to know about any genetic MG predisposition for their children. Precise information about these factors to the patients and to all caretakers during the pregnancy and in the perinatal period should have a supportive and encouraging effect, and also improve the outcome. MG females often have exaggerated worries and postpone or avoid pregnancy unnecessarily.

## Mother's MG

The much higher MG frequency in females than in males during the whole reproductive period strongly indicates that sex hormones play a role in MG pathogenesis. Experimental studies support a role of estrogens and progesterone (9). Thus, both pregnancy, puerperium and lactation would be expected to have the capacity to influence the course of MG. There are several case reports of MG debut during pregnancy, both for AChR- and MuSK antibody-mediated disease. Relative risk of MG onset before, during, and shortly after pregnancy has been calculated in a population-based cohort study combining data from Norway and The Netherlands. 246 women with MG onset at age 15–45 years were included (10). The authors found that the relative risk for onset during pregnancy was not increased. In contrast, this risk increased markedly, with a factor of around five, during the first 6 months postpartum. During the next 6 months, the relative risk normalized. The risk was highest after the first childbirth. Similar results have been reported for other autoimmune disorders such as thyroiditis and rheumatoid arthritis (11). Both hormonal, immunological, and stress mechanisms have been put forward as explanations for MG debut shortly after childbirth.

Established and stable MG can be influenced by pregnancy. Pregnancy is associated with changes in immune and endocrine signaling that can influence autoimmune diseases in general (12). In a series of 69 MG pregnancies, 30% had an exacerbation, 45% had no change, and 25% improved (13). In several similar case series, each with relatively few patients, a deterioration occurred in 35–45% of MG pregnancies (14–18). The rates for exacerbation tended to be higher than for improvement, whereas a substantial proportion remained unchanged. The exacerbations were generally mild to moderate, and myasthenic crisis during pregnancy is rare. Exacerbations occurred more commonly during the first 6 months postpartum than in the pregnancy (17). There were no specific characteristics for the MG patients with exacerbations during pregnancy. Neither previous thymectomy, AChR antibody concentration, nor years since MG debut seemed to be determinants. MG with more severe symptoms before pregnancy usually remained more severe also during this period. More surprisingly, the outcome regarding mother's MG during previous pregnancies did not predict the development next time. This supports the conclusion that pregnancy by itself represents no major risk factor for MG and that non-pregnancy factors are more important both for short-term and long-term MG outcome. In the postpartum period, however, there is an increased risk for both debut of MG and MG deterioration. Among 27 pregnancies either before or during MG with MuSK antibodies, the pregnancy and puerperium did not precipitate or influence mother's muscle weakness (19).

Symptomatic treatment with the acetylcholine esterase inhibitor pyridostigmine is regarded safe and should be continued during pregnancy (20, 21). The drug does not cross placenta in significant amounts. Optimal pyridostigmine treatment is important for most MG women's general health during pregnancy. Some of the reported MG exacerbations during pregnancy is probably due to dose reduction or withdrawal of effective treatments due to fear for harmful effects for the child. Intravenous injections of acetylcholine esterase inhibitor should be avoided during pregnancy as this can lead to increased uterine contractions.

Mycophenolate mofetil, methotrexate and cyclophosphamide are teratogenic immunosuppressive drugs that should not be given to pregnant women (20–22). These drugs should therefore be avoided for all women in reproductive age, at least if there is any chance for pregnancy. Both prednisone/prednisolone and azathioprine are regarded as safe during pregnancy. These are the most common first-line immunosuppressive drug therapies for MG. Rituximab is increasingly used for moderate and severe MG. This is a monoclonal antibody that crosses the placenta. The drug will bind to B-lymphocytes in the developing child and should therefore be avoided the last months before as well as during pregnancy. Newborns of mothers treated with rituximab have transient B-cell depletion (23). This will normalize after 6 months, but it is not known if such children will experience any long-term immunoregulatory complications. Teratogenicity is not a risk for rituximab. Intravenous immunoglobulin and plasma exchange represent safe treatments during pregnancy. Due to convenience and general safety, intravenous immunoglobulin is often the preferred treatment for an MG exacerbation during pregnancy, and for a stable severe or moderately severe MG condition as well. Thymectomy as MG treatment should not be undertaken during pregnancy.

## Pregnancy in MG

MG is not expected to influence fertility. There is an overlap with other autoimmune disorders, and with some that may be associated with female infertility. This has been reported for thyroid disease with reduced thyroid function, SLE, and anti-phospholipid syndrome (24). Autoantibodies *per se* do not seem to be associated with infertility. The commonly used drugs in MG should not reduce fertility. Females with MG tend to have fewer children than healthy women, but this can be explained by other reasons than reduced fertility (8).

Pregnancy is for the large majority of MG females uncomplicated, and MG women should be supported when they wish to have children. However, in a cross-sectional study from Germany, one half of the MG females reported that they had abstained from having a child or further children due to their disease (8). The most common cause was fear of adverse drug effects on the child. The knowledge level was generally low among the MG women. Most pregnancy complications occur with a similar frequency with and without MG, including preeclampsia and eclampsia. However, preterm rupture of amniotic membranes shows an increased frequency, and especially in those with MG deterioration during the pregnancy (15, 25, 26).

Spontaneous abortion may occur with a slightly increased frequency in MG. The exact frequency of miscarriages is difficult to know due to small case series reported, and in addition the possibility of selection bias in the reports. Seven miscarriages among 36 pregnancies were found in a French study (18), 10 among 64 in a similar Italian study (14), 4 among 27 in a Turkish cohort (15), and 5 among 35 in Brazil (16). This indicates a rate of around 15%. This is similar to the miscarriage rate in the general population of 10–20% among women who know they are pregnant. A recent study reported a 24% pregnancy loss rate in females with a spectrum of medical disorders on azathioprine and a 50% risk on mycophenolate mofetil (27).

Folic acid supplement is recommended for MG women in the same way as for other women. The standard recommendation is 400 mg daily before and during pregnancy to reduce the risk of birth defects (28).

## Giving Birth in MG

MG women should be advised to give birth by vaginal delivery, similar to women without MG. However, all case series reports show an increased frequency of Cesarean section. In a national and registry-based Norwegian cohort, 17% of MG females had Cesarean section compared to 8.6% in the total population (25). Both elective and emergency sections were increased. Interestingly, the Cesarean section rate was 15% also in females that had no MG diagnosis when giving birth but had developed overt MG at a later delivery (29). In other MG patient series, the Cesarean section rate is much higher, but with similarly increased rates for the general population. In Taiwan, 45% of MG women had Cesarean section, compared to 37.4% of the general population (30). More than 50 countries in the world have Cesarean section rates above 27% for the total population (31). The British guidelines state that Cesarean section in MG should be performed only for obstetric indications (20). These include prolonged labor with an exhausted mother. Interventions with vacuum or forceps are slightly more common in MG, 9% in MG vs. 6% in the general population in the Norwegian cohort (25).

MG women should continue with their standard drug treatment during the last part of pregnancy and during labor. Epidural analgesia is preferable to general anesthesia whenever possible (20), and is performed in the large majority of those with Cesarean section (16). Most anesthetic drugs are, however, safe in MG. Giving birth at a hospital with experience in neonatal intensive care and with access to a multidisciplinary team involvement by obstetrician, anesthetist, neonatologist, and neurologist is strongly recommended. A protocol with epidural labor analgesia and early use of vacuum extraction for maternal MG has been suggested (32).

## Neonatal Myasthenia

Around 10% of the babies of mothers with MG have a transient muscle weakness. This is due to antibodies against AChR or MuSK that are transported from the mother's circulation, across placenta, and to the fetus (21, 33). In the baby, these antibodies may bind to their respective antigens and induce muscle weakness. If present, the weakness will nearly always appear during the first 24 h after birth. As mother's IgG antibodies are broken down in the baby and gradually disappear, the muscle weakness improves, and normal function is achieved (14). The weakness usually lasts for up to 4 weeks but is most pronounced during the first week.

Typical symptoms are some general hypotonia and poor sucking due to reduced muscle strength. Dysphagia and a weak cry are other possible manifestations. Insufficient respiration, aspiration and pneumonia are rare complications, but make neonatal ward observation necessary for these babies.

In a Norwegian nationwide cohort without selection bias, 5 out of 125 MG babies had definite neonatal myasthenia and another 10 were transferred to a neonatal ward (26). Various case series have reported transient neonatal myasthenia in 4/31, 6/27, 2/30, 1/36, and 5/55 mothers with MG (14–18). This sums up to a frequency of around 10%. The different results can probably best be explained by variation in diagnostic sensitivity for neonatal myasthenia.

Neonatal myasthenia can occur in babies of MG mothers with both AChR and MuSK antibodies, but also in patients without detectable muscle antibodies (34). A large proportion of MG patients where no antibodies can be detected by routine assays, still have such antibodies but with low affinity or in low concentration (35). There is no direct correlation between severity of mother's MG and risk for neonatal myasthenia, nor is there a correlation to antibody concentration in the mother. Transport of IgG across placenta shows individual variation and depends also on properties of the antibodies such as IgG subclass. The serum IgG concentrations in mother and child at delivery are similar, illustrating the efficient transplacental transport during the end of the pregnancy. Epitope specificity of the AChR or MuSK antibody is an important determinant for myasthenic disease, and the configuration and antigenicity of AChR differ between mother and her newborn child (26). Neonatal myasthenia in a previous child increases the risk for the condition in the next ones (36). Previous thymectomy seems to reduce the risk for neonatal myasthenia (37).

Most cases of neonatal myasthenia are so mild that no treatment is needed. Very low doses of the acetylcholine esterase drugs pyridostigmine and neostigmine will improve muscle strength (20). Supportive treatment, for example help with breastfeeding, is important. Treatment with intravenous immunoglobulin or plasma exchange is only very rarely needed.

## Persistent Sequela in the Child

The great majority of children of MG mothers are healthy and with no persistent muscle weakness or motor disabilities. This is true also for those with transient neonatal myasthenia. IgG transport across placenta does not appear until pregnancy week 13, after the organ-forming period. In pregnancy week 17–22, the IgG concentration in the child is still only 5–10% of that in the mother (38, 39).

Arthrogryposis with skeletal abnormalities and joint contractures is a rare condition but has increased frequency in children of MG mothers (40). Five out of 127 such children (3.9%) in the Norwegian national cohort had such malformations (25). No cases of congenital malformations have been reported in other case series with 26 and 30 children (16, 17). MG in mother does not seem to be a major causative risk factor for arthrogryposis (41). As for neonatal myasthenia, a previous child with arthrogryposis represents a definite risk factor in the next pregnancy (26). Such women should be treated with intravenous immunoglobulin or plasma exchange in all later pregnancies. The cause of arthrogryposis is restricted fetal movements *in utero*. When mother has MG the movement restriction is due to mother's IgG antibodies binding to fetal type AChR with gamma subunits. Arthrogryposis can occur in babies of mothers also with only mild MG. Fetal movements should be monitored as accurately as possible (40) in all women with MG, as there is effective treatment to inhibit arthrogryposis to develop in MG mothers.

In rare, single cases, a permanent muscle weakness has been reported in children of MG mothers (42, 43). This weakness can be generalized or isolated, for example as a facial paresis. This is not a fluctuating condition due to persistent antibodies, but rather a permanent change in the postsynaptic membrane induced by mother's AChR antibodies during fetal life. Such a fetal AChR inactivation syndrome has been reported in eight children from four families (42).

## Breastfeeding

Breastfeeding should be encouraged for MG mothers (20, 21). This is true both for those with AChR and MuSK antibodies. Maternal lgG levels in milk comprise only 2% of that in serum. In humans, breast milk does not represent a source for immunity transfer from mother to child. Breastfeeding is recommended also for babies with neonatal myasthenia. Being breastfed has many advantages, including a reduced risk for autoimmune disease later in life (44).

Breastfeeding is not known to influence mother's MG. There is an increased risk for worsening of MG in the puerperium, similar to other autoimmune disorders. Boldingh et al. found that debut of MG in the postpartum period was more common in The Netherlands than in Norway, and they speculated that the much higher frequency of prolonged breastfeeding in Norway might have a protective role (10).

Breastfeeding is advised against in MG mothers with ongoing treatment with cyclophosphamide, mycophenolate mofetil or methotrexate (20). The reason is the teratogenic potential of these drugs. Cyclophosphamide is excreted into breast milk. Maternal treatment with pyridostigmine, prednisolone/prednisone, or azathioprine represents no contraindication for breastfeeding. Transfer of these drugs and their metabolites into breast milk is minimal. Breastfeeding is most probably safe also for treatment with rituximab, cyclosporine, and tacrolimus. The concentration of rituximab in breast milk is 200 times less than in serum (45). Intravenous immunoglobulin or plasma exchange can be used for MG exacerbations in the postpartum period irrespective of breastfeeding or not. Breastfeeding should be encouraged for women on treatment with monoclonal antibodies, and at the same time register outcome (46).

## Comorbidities

MG women have an increased frequency of all other autoimmune disorders (47). Such disorders need to be taken into consideration for women before and during pregnancy, both their clinical manifestations and their treatment. In a minority of young women, the MG is caused by a thymoma. Most MG-related thymomas should not influence pregnancy, but in rare cases either thymoma treatment or non-MG thymoma-associated autoimmune disease may be of significance. Infections should always be treated actively in MG patients, and with specific considerations regarding choice of anti-infectious drugs (48).

## Conclusion

MG women with a child wish should be supported and encouraged. Pregnancy and childbirth have similar complication rates as for the non-MG population. Optimal drug treatment for MG should be continued. Vaginal delivery is recommended, and indications for Cesarean section are obstetrical and the same as for non-MG women. Breastfeeding is safe and should be supported. However, there are a few important warnings. Mycophenolate mofetil, methotrexate and cyclophosphamide should not be given to any females that may become pregnant as these drugs have a teratogenic potential. Rituximab should be stopped some months before a pregnancy. MG women should always give birth at a hospital with intensive care services for the newborn, as 10% of the babies have transient neonatal myasthenia. All babies by MG mothers should be observed in hospital for at least 48 h. Correct information to all females in reproductive age is important. Obstetrical and neurological follow-up during pregnancy makes a difference. Many MG women have exaggerated worries and practice unnecessary limitations or restrictions regarding pregnancy.

## Author Contributions

The author confirms being the sole contributor of this work and has approved it for publication.

## Conflict of Interest

NG has received speaker's or committee work honoraria from Argenx, Ra Pharma, UCB, Octapharma, Alexion, Merck.
